# Disgust Sensitivity Among Women During the COVID-19 Outbreak

**DOI:** 10.3389/fpsyg.2021.622634

**Published:** 2021-03-23

**Authors:** Karolina Miłkowska, Andrzej Galbarczyk, Magdalena Mijas, Grazyna Jasienska

**Affiliations:** Department of Environmental Health, Faculty of Health Science, Jagiellonian University Medical College, Krakow, Poland

**Keywords:** COVID-19 pandemic, aversion, pathogen stress, evolutionary psychology, behavioral immune system

## Abstract

The emotion of disgust is suggested to be an adaptation that evolved to keep us away from sources of infection. Therefore, individuals from populations with greater pathogen stress should have a greater disgust sensitivity. However, current evidence for a positive relationship between disgust sensitivity and the intensity of infectious diseases in the environment is limited. We tested whether disgust and contamination sensitivity changed in response to the COVID-19 pandemic. Disgust was assessed in 984 women in 2017 (before pandemic) and 633 women in 2020 (during pandemic) by a set of photographs depicting sources of infection and Pathogen and Moral of Three-Domain Disgust Scale. Further, contamination sensitivity among participants in two waves was measured by Contamination Obsessions and Washing Compulsions Subscale of Padua Inventory. State anxiety was measured with the Polish adaptation of the State-Trait Anxiety Inventory (STAI) only during the second wave of data collection. Women from the COVID-19 pandemic group assessed the photographs depicting sources of infection as more disgusting, scoring higher on Padua Inventory, but lower on Moral Disgust Domain as compared to women from before the pandemic. In addition, anxiety levels during pandemic positively correlated with scores from Pathogen Disgust Domain, Padua Inventory, and the ratings of the photographs. The participants of the study scored higher in state anxiety than the norms determined for the Polish population. Summarizing, we present evidence for differences in individual levels of disgust sensitivity in relation to pathogen stress, supporting the idea that disgust evolved to serve as protection from pathogens.

## Introduction

Disgust, a universal human emotion, is elicited by a number of factors, including the sources of potential pathogens, such as bodily fluids, people with visible symptoms of disease, dirty environments, rotting food, certain animals, or the violations of moral norms, and antisocial behaviors, including cheating and stealing (Darwin, 1872; Brown, 1991; Curtis and Biran, 2001). The facial expression of disgust (wrinkling of the nose, pulling down the corners of the mouth) is recognizable across cultures (Mesquita and Frijda, 1992). Disgust may be accompanied by strong physical reactions, such as galvanic skin responses, lowered blood pressure, and nausea (Rozin et al., 1993). Pathogen disgust motivates the avoidance of infectious pathogens and is the first line of defense against pathogens (Wicker et al., 2003; Oaten et al., 2009; Stevenson et al., 2009, 2011; Tybur et al., 2009; Curtis et al., 2011). Moral disgust, on the other hand, serves the purpose of avoiding social norm violations (Tybur et al., 2009; Curtis, 2011).

People vary in the degree to which they experience disgust toward pathogens cues. Research concerning behavioral immune system has suggested that greater infection-avoiding behavior and attitudes might be triggered by the presence of infection cues and by one's intrinsic vulnerability to infection. It has been suggested that pathogen avoidance motivation may promote health and longevity by allowing for lower levels of non-targeted inflammation without an increase in infection risk (Gassen et al., 2018). Hence, disgust may be one mechanism that helps people effectively manage such threats, where highly disease-avoidant people bear lower infection costs (e.g., Gassen et al., 2018; Cepon-Robins et al., 2021).

Another line of inquiry suggests that disgust sensitivity is shaped by earlier exposures to pathogens (Tybur et al., 2018), and that it is higher in people who are relatively more vulnerable to infectious diseases (Schaller, 2011). Studies have shown higher anxiety among patients with rheumatoid arthritis toward infection-risky behaviors (Oaten et al., 2017) and heightened attention to and avoidance of individuals displaying disease cues among recently ill people (Miller and Maner, 2011). Moreover, it has been suggested that not only decreased ability to avoid illness might alter disgust sensitivity, but priming disease cues also might upregulate disgust (e.g., Curtis et al., 2011). Thus, people might functionally upregulate disgust during the pandemic to manage ongoing infection threats (Skolnick and Dzkoto, 2013; Ackerman et al., 2021; Stevenson et al., 2021). Summarizing, a degree to which people are disgusted by pathogen cues should depend on pathogen stress, and, consequently, on the risk of getting infected.

However, current evidence for the positive relationship between pathogen stress and disgust level in humans is limited. Attempts to compare disgust across a number of nations that vary in pathogen stress have led to inconclusive results (Curtis et al., 2011; Skolnick and Dzkoto, 2013; Tybur et al., 2016; Ackerman et al., 2021; Stevenson et al., 2021). Based on this there might be no relationship between current infection burden and pathogen disgust (Curtis et al., 2011; Tybur et al., 2016). Comparing different nations, however, is biased due to the fact that disgust sensitivity may be influenced by culture-specific factors, for example, cultural ideas of purity and pollution. Other attempts to demonstrate that disgust sensitivity and pathogens are connected focused on differences in individual disgust sensitivity and illness frequency (Stevenson et al., 2009), history of infectious diseases in childhood (de Barra et al., 2014), or general health (Prokop et al., 2010; Prokop and Fančovičová, 2011). However, the results of these studies fell short of being conclusive. For example, no effect of illness recency on attentional bias for disfigured faces was found in a replication of Miller and Maner's (2011) study (Tybur et al., 2020). It has been also shown that decreased ability to avoid infections downregulates rather than upregulates disgust (Bartres and Perrett, 2020; Cepon-Robins et al., 2021).

Since December 2019, an outbreak of respiratory disease caused by a new strain of coronavirus (SARS-CoV-2) has spread rapidly throughout the world, dramatically increasing pathogen stress in many countries. The dramatic change in the prevalence and virulence of pathogens in the environment during the COVID-19 pandemic created unique conditions for testing the relationship between the level of disgust and pathogen stress within a single population. If disgust, indeed, serves as a first line of defense against pathogens, people should show greater pathogen disgust during a pandemic than during a time of lower pathogen stress. To this date only Stevenson et al. (2021) have shown that university students during Australia's COVID-19 pandemic lockdown period reported higher disgust sensitivity, while comparing to earlier student cohorts. In this study, we aim to test whether there is a difference in disgust sensitivity level between the data collected in year 2017—before the COVID-19 pandemic—and during the pandemic in 2020.

## Materials and Methods

### Participants

In 2017 (before the COVID-19 pandemic) we recruited 984 women and in 2020 (during the COVID-19 pandemic) −633 women. All the participants were Poles, aged 18–45. All women completed at least one questionnaire on disgust level. The first wave of data was collected in 2017 as part of a study that examined relations between disgust sensitivity and menstrual cycle phases in women (Miłkowska et al., 2019). In March 2020, Polish government introduced special restrictions due to the growing number of COVID-19 cases, including social distancing, closing of state borders, schools, cinemas, most stores, and restricting the number of people in churches. The 2020 sample was selected to match the first round of data collection, both in terms of the methods used and the sample size. A sensitivity analysis revealed that the sample size of 1,617 allowed us to detect a small effect size of $\eta_{\text{p}}^{2}=0.004$.

### Procedure

The research was conducted in two rounds: before the COVID-19 pandemic in May and June 2017, and during the COVID-19 pandemic in April and May 2020. The data was collected using the same protocol during both waves. Information about the study and invitation to participate were published in social media e.g., on Facebook through the fanpages of Polish women's magazines, e.g., Women Health's and advertised as a “Study on disgust in women.” Women were not compensated for participation. The surveys were available in the Polish language for participants at: www.qualtrics.com (before the COVID-19 pandemic) and at: www.limesurvey.org (during the COVID-19 pandemic).

The first part of the survey included questions about health and selected demographic information, and the second part of the survey consisted of questions about disgust sensitivity and experiencing anxiety. Informed consent was obtained from all participants of the study.

### Measures

#### Disgust

Disgust sensitivity in women was assessed by set of photographs depicting sources of infection (Curtis et al., 2004) and two of three domains of Three-Domain Disgust Scale (Pathogen Disgust and Moral Disgust Domains; Tybur et al., 2009).

The women assessed the intensity of their disgust feelings while looking at each of 20 photographs (Curtis et al., 2004). Women assessed each picture on a 5-point Likert-type scale where 1 stood for “not disgusted” and 5 for “very disgusted.” The analyses included ratings of only seven photos that showed potential source of infections, such as: a person looking feverish and spotty-faced, inside of a crowded underground train, a skin lesion with pus and inflammation, a plate of viscous liquid resembling bodily fluids, stained towel with reddish-yellow bodily secretions, louse, and ascaris worms.

Additionally, the participants answered seven questions from Pathogen Disgust Domain and seven from Moral Disgust Domain of TDDS (Tybur et al., 2009). The third domain—Sexual Disgust Domain—was not included in the first wave of data collection, as it is usually not used in research on disgust sensitivity across menstrual cycle (Zelazniewicz et al., 2016; Miłkowska et al., 2019). The questions in Pathogen and Moral Domains concerned the level of disgust toward hypothetical situations e.g., seeing a cockroach run across the floor, shaking hands with a stranger who has sweaty palms, shoplifting a candy bar from a convenience store, or intentionally lying during a business transaction. The items of questionnaire were scored on a 7-point Likert-type scale ranging from “not at all disgusting” (1) to “extremely disgusting” (7). The higher a woman scored in both questionnaires, the higher disgust sensitivity she exhibited.

#### Contamination Sensitivity

The level of contamination sensitivity was also measured twice among two groups of women. The participants answered questions from Contamination Obsessions and Washing Compulsions Subscale of Padua Inventory—Washington State University Revision (Burns et al., 1996). We used a 5-point Likert-type scale ranging from “not at all” (1) and “very much” (5) e.g., “I feel my hands are dirty when I touch money.”

#### Anxiety

State anxiety was measured only during the second wave of data collection with the Polish adaptation of the State-Trait Anxiety Inventory's (STAI) subscale dedicated to measure anxiety defined as subjective transitory feelings of angst and tension (Spielberger et al., 1970; Sosnowski et al., 2011). The state anxiety STAI subscale consists of 20 items (e.g., I feel calm; I feel secure; I feel tense) rated on a Likert-type scale with higher scores indicating greater anxiety level.

### Statistical Methods

Preliminary analyses compared groups of women participating in the study before and during the COVID-19 pandemic in age (by the *t*-test) and in occupation status and long-term health problems (by the chi-squared test). The pre-pandemic and pandemic groups did not differ with regard to occupation status [$\chi_{\left(1\right)}^{2}=0.640$, *p* = 0.423], belonging to professions connected to potential disgust elicitors (i.e., involving contact with dirt, animals, body secretions, animal, or human tissue) [$\chi_{\left(1\right)}^{2}=$ 0.039, *p* = 0.843], or having long-term health problems (lasting longer than 12 months) [$\chi_{\left(1\right)}^{2}=$ 0.021, *p* = 0.883]. The groups of women differed in mean age: those who took part in the study in 2020 were about 1 year older (mean = 27.7, *SD* = 6.60) than those from the 2017 group (mean = 26.6, *SD* = 5.95) [*t*_(1615)_ = −3.261, *p* < 0.001] (Table 1).

**Table 1:** The comparison of women from COVID-19 pandemic and pre-pandemic groups with respect to their age (*t*-test), occupation, profession connected to disgust elicitors, and long-term health problems (chi-squared).

	**Before COVID-19**		**During COVID-19**				
**Variable**	**Mean**	**SD**	**Mean**	***SD***	***t***	***df***	***p***
Age	26.6	5.95	27.7	6.60	**−3.261**	**1615**	**<0.001**
	***n***	**%**	***n***	**%**	***χ***^**2**^	**df**	**p**
Occupation							
Yes	564	57.32	350	55.29	0.640	1	0.423
No	420	42.68	283	44.71			
Profession connected to disgust elicitors^*^							
Yes	118	11.99	78	12.32	0.039	1	0.843
No	866	88.01	555	87.68			
Long-term health problems							
Yes	301	30.84	194	31.19	0.021	1	0.883
No	675	69.16	428	68.81			

* *Profession involving contact with dirt, animals, body secretions, and animal, or human tissue. Statistically significant results are in bold*.

Since the groups of women slightly differed in age, all the differences in disgust sensitivity between them were subjected to analyses of covariance (ANCOVA), with age as a potential confounder. Moreover, a linear regression was used to analyze the correlation between disgust sensitivity and STAI. State-Trait Anxiety Inventory scores were also converted to standardized “Standard Ten” (sten) scores in order to compare anxiety levels in study sample with the norms determined for the Polish population. A sten score reflects individual's position relative to other people from the population of reference. “Standard Ten” scale ranges from 1 to 10 with a mean value of 5.5 and standard deviation of 2. In psychometric assessments calculating raw questionnaire scores to sten scores is a standard practice. All statistical analyses were performed in STATISTICA 13.3 and JASP (Version 0.11.1; JASP Team, 2019).

## Results

As compared to the participants from the pre-pandemic group the women who took part in the study during the COVID-19 pandemic assessed the photographs depicting the sources of infection as more disgusting [*F*_(1,1537)_ = 433.82, *p* < 0.001], and scored higher on the Contamination Obsessions and Washing Compulsions Subscale of Padua Inventory [*F*_(1,1576)_ = 38.42, *p* < 0.001]. Moreover, they had lower scores in Moral Disgust Domain than the pre-pandemic group [*F*_(1,1614)_ = 6.28, *p* = 0.012]. There were no statistically significant differences in Pathogen Disgust Domain among these two groups [*F*_(1,1614)_ = 0.551, *p* = 0.458] (Table 2).

**Table 2:** Disgust sensitivity among women—the comparison of groups of women from COVID-19 pandemic and pre-pandemic groups after controlling for age.

		***n***	**Adj mean**	***SE***	***F***	***P***	***$\eta_{p}^{2}$***
Photographic stimuli (mean score)	Before COVID-19	942	3.33	0.02	**433.82**	**<0.001**	**0.220**
	During COVID-19	597	4.19	0.04			
Padua inventory	Before COVID-19	966	2.37	0.03	**38.417**	**<0.001**	**0.024**
	During COVID-19	613	2.61	0.04			
Moral disgust	Before COVID-19	942	5.22	0.04	**6.276**	**0.012**	**0.004**
	During COVID-19	597	5.11	0.05			
Pathogen disgust	Before COVID-19	984	4.71	0.04	0.551	0.458	<0.001
	During COVID-19	633	4.66	0.04			

*Statistically significant results are in bold*.

Among women participating in the study during the COVID-19 pandemic, anxiety (measured by STAI questionnaire) positively correlated with scores from Pathogen Disgust Domain (*β* = 0.10, *p* = 0.011), the Padua Inventory (*β* = 0.16, *p* < 0.001), and the ratings of the photographs (*β* = 0.13, *p* < 0.001). However, the correlation between scores from STAI and Moral Disgust Domain was not statistically significant (*β* = 0.03, *p* = 0.431; Table 3). The participants of the study also scored 0.36 stens higher in state anxiety than the population of reference, and this difference was statistically significant (*t* = 3.33, *df* = 583, *p* < 0.001).

**Table 3:** Relationship between disgust sensitivity and state anxiety (State-Trait Anxiety Inventory).

	***n***	**β**	***SE***	***p***
Photographic stimuli (mean score)	584	0.13	0.003	**<0.001**
Padua inventory	584	0.16	0.04	**<0.001**
Moral disgust	584	0.03	0.79	0.431
Pathogen disgust	584	0.10	0.03	**0.012**

*Statistically significant results are in bold*.

## Discussion

Our results of the comparison of disgust sensitivity and contamination sensitivity between two groups of women characterized by similar demographics during time periods characterized by a different pathogen stress level supports the idea that disgust as a behavioral adaptation is the first psychobehavioral line of defense against pathogens. As compared to the women from before the COVID-19 outbreak the group from the time of the COVID-19 pandemic assessed the photographs of sources of infection as more disgusting, and scored higher on the Contamination Obsessions and Washing Compulsions Subscale of Padua Inventory, but not on Pathogen Disgust of Three-Domain Disgust Scale. The observed difference was most pronounced in the case of response to visual stimuli (effect size $\eta_{\text{p}}^{2}=$ 0.220). Significantly, some researchers have suggested that visual methods of measurement (including measurement of reaction time) provide the most objective method of assessing the mechanisms of pathogen disgust (Miller and Maner, 2011; Ersche et al., 2014). Hence, as we hypothesized, when the environment becomes more dangerous through increased exposure to infections, people enhance their disgust sensitivity.

Our results are partially consistent with Stevenson, Saluja and Case (2021) study on the impact of the COVID-19 pandemic on disgust sensitivity, using a different population and measures. Students during Australia's lockdown period of the COVID-19 pandemic reported overall higher levels of disgust sensitivity and higher scores for Core Disgust subscale from revised version of Disgust Scale (Olatunji et al., 2009), which is most similar to the Pathogen Disgust of Three-Domain Disgust Scale used in our study. Interestingly, while Stevenson et al. (2021) found evidence for differences in self-reported disgust scale answers in a mixed-sex population of college students in Australia, we found evidence for a difference in disgust sensitivity using a naturalistic measure (the photographs of infection sources) but not a self-report scale with a sample of women from Poland. Stevenson et al. (2021) also provided some evidence of an increase in germ aversion and an increase in hand and food-related hygiene.

Further, our results are also in line with Skolnick and Dzkoto (2013), who found a higher level of disgust sensitivity in a country with relatively high pathogen stress (i.e., Ghana), as compared to a country of relatively low parasite stress (i.e., USA). However, other studies have shown a lack of differences in the level of disgust among participants from countries with different infectious disease rates (Tybur et al., 2016), and in disgust ratings of photographs across nine world regions (e.g., Europe, the Far East, North America, Latin America, the Indian Subcontinent, and the Eastern Block; Curtis et al., 2011). However, comparing disgust sensitivity in different nations can be problematic not only due to cross-cultural variation in food preferences, hygiene norms, and taboos (e.g., Sherman and Billing, 1999; Navarrete and Fessler, 2003), but also due to the population's variation in genetic mutations conferring resistance to infectious diseases (e.g., Prugnolle et al., 2005; Fumagalli et al., 2009).

The studies that focused on differences in individual levels of disgust sensitivity in relation to health status in a single population are limited, and provide inconclusive results. On the one hand, stronger emotions, which should protect against infections, correlated with better health. Higher disgust sensitivity was associated with fewer recent infections (Stevenson et al., 2009), lower infection burden (e.g., Gassen et al., 2018; Cepon-Robins et al., 2021), and pathogen avoidance behaviors were more frequently reported by healthy people (Prokop and Fančovičová, 2011). Moreover, a childhood illness and, to a lesser extent, a recent illness were associated with perceived infectability (Makhanova et al., in press). However, other studies indicated that higher fear and disgust were associated with worse health. For example, higher fear of disease-relevant animals was found in participants with lower self-perceived health (Prokop et al., 2010); the level of disgust with ectoparasites positively correlated with a total number of reported illnesses (Prokop and Fančovičová, 2011); and higher contamination sensitivity was associated with more frequent infectious illnesses (Stevenson et al., 2009). Furthermore, some studies failed to find any relationships between disgust sensitivity and health. A study by de Barra et al. (2014) showed a lack of relationship between having more infectious diseases in childhood and greater adult disgust sensitivity. Oaten et al. (2017) demonstrated that disgust sensitivity did not differ between people with rheumatoid arthritis (increasing the risk of infection-related morbidity and mortality) and healthy controls. It should be noted, however, that none of these studies addressed actual pathogen stress exposure.

Another finding of our study is related to the Moral Disgust Domain. During the COVID-19 pandemic the women had lower moral disgust scores than before the pandemic. It has been suggested that many traditions, rituals, religious beliefs, and moral norms historically helped to prevent infectious diseases (Fabrega, 1997). Therefore, people under high pathogen stress should respond especially harshly to norm violations. For example, it has been shown that individuals in nations with greater parasite stress reported stronger adherence to traditional norms (Tybur et al., 2016). At the individual level, some studies suggested that experienced disgust triggered by, for example, exposure to a bad smell (“fart spray”; Schnall et al., 2008), drinking bitter liquid (Eskine et al., 2011), or watching a revolting clip (the toilet scene from *Trainspotting*; Schnall et al., 2008) can increase the severity of moral judgments. However, other studies failed to replicate these results (i.e., Schnall et al., 2008; Ugazio et al., 2012; Johnson et al., 2014). Therefore, the role of disgust, triggered by potential infection sources, for moral judgment is still unclear (for review see Landy and Goodwin, 2015). Horberg et al. (2009) suggested that higher disgust sensitivity might be positively related to stronger condemnation of behaviors violating purity (consensual incest; having sex with a dead chicken prior to consuming it), but not with punishment of justice transgressions (not returning an important library book; interrupting meetings to ask for small favors).

It should be noted that the Moral Domain in the Three-Domain Disgust Scale used in our study does not pertain to any purity transgressions (Tybur et al., 2009). The questions relate only to justice and loyalty validation (e.g., deceiving a friend, stealing from a neighbor, lying during a business transaction, shoplifting a candy bar, forging someone's signature on a legal document). Thus, it is possible that in our study the women who participated during the COVID-19 pandemic were less disgusted by behaviors that in a time of reduced wages, supply shortages, and economic uncertainty might help in self-preservation and the assurance of financial security.

We also observed significant associations between the scores of state anxiety and the Pathogen Disgust in the Three-Domain Disgust Scale, the Contamination Obsessions and Washing Compulsions Subscale of the Padua Inventory, and the ratings of photographs of sources of infection. These results are consistent with studies reporting associations between disgust sensitivity and anxiety related to potential health hazards (Fan and Olatunji, 2013). Further, as Stevenson et al. (2021) suggested, the level of threat that people perceive during the COVID-19 pandemic might be far higher than normal, which could in turn increase the intensity of disgust sensitivity. For instance, in a study on psychological processes associated with the Ebola outbreak in the 2014, the fear of the disease was associated with increased general distress, body vigilance, and disgust sensitivity (Blakey et al., 2015). We did not, however, observe similar correlations between state anxiety and moral disgust, which suggests that moral disgust is associated with different psychological mechanisms, and is to a lesser extent driven by anxiety.

One of the limitations of the study design was the lack of the possibility to compare the disgust sensitivity of the same women before and during the COVID-19 pandemic. We are fully aware of the bias related to between-subject design, including the confounding effect of inter-individual differences. However, such data is much more difficult to collect, especially in a pandemic context. In contrast to previous studies we did not compare different nations (Curtis et al., 2011; Skolnick and Dzkoto, 2013; Tybur et al., 2016), but two large groups of women from the same country under changed environmental conditions. The groups did not differ in any factors that could influence their perception of disgust (i.e., occupational status, long-term health problems, or belonging to professions connected to potential disgust elicitors). A further limitation is that we compared ratings only in groups of women. However, as reported before, disgust sensitivity varies consistently between men and women, with higher scores on measures of disgust sensitivity in women than men (Haidt et al., 1994; Rozin et al., 1999; Curtis et al., 2004). Moreover, in our study we tested only differences in disgust sensitivity and contamination sensitivity. Hence, future studies concerning this topic would benefit from analyzing both women and men, using a longitudinal study design, and including a wider range of emotions.

In our study it was documented that the participants from the pandemic group assessed the photographs of infection sources as more disgusting, but they did not show any increase in the Pathogen Disgust of the Three-Domain Disgust Scale. This lack of differences might be caused by the characteristics of the questionnaire, which was criticized by Fleischman and Fessler (2018), and by Tybur et al. (2016) as potentially insensitive in pathogen avoidance motivations. Self-reported disgust with graphic visual images containing disease cues has been proposed as a more sensitive and accurate measure of pathogen disgust sensitivity than a self-reported disgust for text-only questionnaire items (Fleischman and Fessler, 2018).

Summarizing, we present a comparison of disgust level and contamination sensitivity in two groups of women characterized by similar demographics during two time points when pathogen stress varied. The outbreak of the COVID-19 pandemic provided an opportunity to compare the population's samples from two different pathogenic environments. Our results, indicating higher level of disgust sensitivity during the COVID-19 pandemic compared to pre-pandemic period, support the idea that disgust evolved to serve as a form of protection from pathogens.

## Data Availability Statement

The raw data supporting the conclusions of this article will be made available by the authors, without undue reservation.

## Ethics Statement

The studies involving human participants were reviewed and approved by Jagiellonian University Ethics Committee. The patients/participants provided their written informed consent to participate in this study.

## Author Contributions

KM, AG, and GJ designed the study. KM collected the data. KM, MM, and AG performed statistical analyses. All authors participated in writing a first draft of the paper, editing the manuscript, contributed to the article, and approved the submitted version.

## Conflict of Interest

The authors declare that the research was conducted in the absence of any commercial or financial relationships that could be construed as a potential conflict of interest.

## Abbreviations

STAI: State-Trait Anxiety Inventory.
